# A System Pharmacology Multi-Omics Approach toward Uncontrolled Pediatric Asthma

**DOI:** 10.3390/jpm11060484

**Published:** 2021-05-28

**Authors:** Mahmoud I. Abdel-Aziz, Anne H. Neerincx, Susanne J. H. Vijverberg, Simone Hashimoto, Paul Brinkman, Mario Gorenjak, Antoaneta A. Toncheva, Susanne Harner, Susanne Brandstetter, Christine Wolff, Javier Perez-Garcia, Anna M. Hedman, Catarina Almqvist, Paula Corcuera-Elosegui, Javier Korta-Murua, Olaia Sardón-Prado, Maria Pino-Yanes, Uroš Potočnik, Michael Kabesch, Aletta D. Kraneveld, Anke H. Maitland-van der Zee, on behalf of the SysPharmPediA Consortium

**Affiliations:** 1 Department of Respiratory Medicine, Amsterdam UMC, University of Amsterdam, 1105 AZ Amsterdam, The Netherlands; m.i.ibrahim@amsterdamumc.nl (M.I.A.-A.); a.vanstuyvenbergneerincx@amsterdamumc.nl (A.H.N.); s.j.vijverberg@amsterdamumc.nl (S.J.H.V.); s.hashimoto@amsterdamumc.nl (S.H.); p.brinkman@amsterdamumc.nl (P.B.); 2 Department of Clinical Pharmacy, Faculty of Pharmacy, Assiut University, 71526 Assiut, Egypt; 3 Department of Pediatric Respiratory Medicine, Emma Children’s Hospital, Amsterdam UMC, 1105 AZ Amsterdam, The Netherlands; 4 Center for Human Molecular Genetics and Pharmacogenomics, Faculty of Medicine, University of Maribor, 2000 Maribor, Slovenia; mario.gorenjak@um.si (M.G.); uros.potocnik@um.si (U.P.); 5 Department of Pediatric Pneumology and Allergy, University Children’s Hospital Regensburg (KUNO) at the Hospital St. Hedwig of the Order of St. John, University of Regensburg, D-93049 Regensburg, Germany; Antoaneta.Toncheva@klinik.uni-regensburg.de (A.A.T.); Susanne.Harner@barmherzige-regensburg.de (S.H.); Michael.Kabesch@barmherzige-regensburg.de (M.K.); 6 Science and Development Campus Regensburg (WECARE), University Children’s Hospital Regensburg (KUNO) at the Hospital St. Hedwig of the Order of St. John, University of Regensburg, D-93049 Regensburg, Germany; Susanne.Brandstetter@barmherzige-regensburg.de (S.B.); Christine.Wolff@barmherzige-regensburg.de (C.W.); 7 Genomics and Health Group, Department of Biochemistry, Microbiology, Cell Biology and Genetics, Universidad de La Laguna, 38200 San Cristóbal de La Laguna, Spain; jpegarci@ull.edu.es (J.P.-G.); mdelpino@ull.edu.es (M.P.-Y.); 8 Department of Medical Epidemiology and Biostatistics, Karolinska Institutet, 171 77 Stockholm, Sweden; anna.hedman@ki.se (A.M.H.); Catarina.Almqvist@ki.se (C.A.); 9 Pediatric Allergy and Pulmonology Unit, Astrid Lindgren Children’s Hospital, Karolinska University Hospital, 171 77 Stockholm, Sweden; 10 Division of Pediatric Respiratory Medicine, Hospital Universitario Donostia, 20014 San Sebastián, Spain; PAULA.CORCUERAELOSEGUI@osakidetza.eus (P.C.-E.); JOSEJAVIER.KORTAMURUA@osakidetza.eus (J.K.-M.); OLAIA.SARDONPRADO@osakidetza.eus (O.S.-P.); 11 Department of Pediatrics, University of the Basque Country (UPV/EHU), 20014 San Sebastián, Spain; 12 CIBER de Enfermedades Respiratorias, Instituto de Salud Carlos III, 28029 Madrid, Spain; 13 Instituto de Tecnologías Biomédicas (ITB), Universidad de La Laguna, 38200 San Cristóbal de La Laguna, Spain; 14 Laboratory for Biochemistry, Molecular Biology and Genomics, Faculty of Chemistry and Chemical Engineering, University of Maribor, 2000 Maribor, Slovenia; 15 Division of Pharmacology, Utrecht Institute for Pharmaceutical Sciences, Faculty of Science, Utrecht University, 3584 CG Utrecht, The Netherlands; A.D.Kraneveld@uu.nl

**Keywords:** pediatric asthma, uncontrolled asthma, omics, systems medicine

## Abstract

There is a clinical need to identify children with poor asthma control as early as possible, to optimize treatment and/or to find therapeutic alternatives. Here, we present the “Systems Pharmacology Approach to Uncontrolled Pediatric Asthma” (SysPharmPediA) study, which aims to establish a pediatric cohort of moderate-to-severe uncontrolled and controlled patients with asthma, to investigate pathophysiological mechanisms underlying uncontrolled moderate-to-severe asthma in children on maintenance treatment, using a multi-omics systems medicine approach. In this multicenter observational case–control study, moderate-to-severe asthmatic children (age; 6–17 years) were included from four European countries (Netherlands, Germany, Spain, and Slovenia). Subjects were classified based on asthma control and number of exacerbations. Demographics, current and past patient/family history, and clinical characteristics were collected. In addition, systems-wide omics layers, including epi(genomics), transcriptomics, microbiome, proteomics, and metabolomics were evaluated from multiple samples. In all, 145 children were included in this cohort, 91 with uncontrolled (median age = 12 years, 43% females) and 54 with controlled asthma (median age = 11.7 years, 37% females). The two groups did not show statistically significant differences in age, sex, and body mass index z-score distribution. Comprehensive information and diverse noninvasive biosampling procedures for various omics analyses will provide the opportunity to delineate underlying pathophysiological mechanisms of moderate-to-severe uncontrolled pediatric asthma. This eventually might reveal novel biomarkers, which could potentially be used for noninvasive personalized diagnostics and/or treatment.

## 1. Introduction

Asthma is a chronic respiratory disease characterized by shortness of breath, airway inflammation, and airway hyperresponsiveness. It is a heterogeneous, multifaceted disease with variable severity and treatment response. Asthma affects approximately 350 million people worldwide across different ethnicities and age groups [1], being the most common chronic disease in children [2]. Majority of the asthmatic children respond well to standard therapy with inhaled corticosteroids (ICS), which is considered generally safe for use. However, its long-term use at high dosage is of concern among physicians and parents due to its potential side effects, such as growth retardation [3]. Moreover, despite treatment with high doses of ICS, 2–10% of asthmatic children are not well controlled, experiencing severe symptoms, frequent asthma attacks/exacerbations, and poor quality of life. Furthermore, these children are at a higher risk of adverse effects from high doses of medications [4,5]. Therefore, this severe form of asthma presents substantial health, financial, and psychological burdens on the patients, their families, and healthcare communities [5,6].

Nowadays, childhood asthma is still treated using a “one size fits all” trial and error approach, which is not optimal to achieve maximal control, particularly for severe childhood asthmatics. Childhood asthma is now recognized as consisting of different subtypes with variable clinical manifestations (phenotypes) [7]. By understanding the underlying biological mechanisms driving those phenotypes (endotyping) [7], better diagnostic biomarkers and/or therapeutic options can be personalized to the patients (a treatable mechanism approach) [8]. Hence, a paradigm shift on how asthma is diagnosed and treated traditionally becomes a necessity in the era of personalized medicine. Advances in high-throughput omics technologies have allowed the mapping of thousands of biological molecules within the human body. Different omics analyses, such as (epi)genomics, transcriptomics, proteomics, metabolomics (including breathomics), and microbiomics have shown potential in identifying asthma phenotypes and may serve as unique (molecular) fingerprints for each patient or group of patients. Integrating the information from multi-omics platforms in a systems medicine approach allows better molecular classification of patients (hand printing) and refining asthma phenotypes [9]. However, multi-omics projects in asthma are challenging in terms of costs and required computational and human resources. Therefore, their success requires the coordination and collaboration of diverse research groups from different disciplines in an international multicenter approach. Due to the demanding nature, multi-omics projects in asthma are scarce but have proven value in the comprehensive evaluation of molecular processes in asthma pathogenesis [9]. Successful examples of asthma multi-omics projects in children are the Unbiased Biomarkers in Prediction of Respiratory Disease Outcomes (U-BIOPRED) [10] and Severe Asthma Research Program (SARP) [11,12]. These projects have shown promising potential in revealing asthma phenotypes and delineating some potential endotypes [9].

The Systems Pharmacology Approach to Uncontrolled Pediatric Asthma (SysPharmPediA) cohort is a multicenter pan-European study aiming to apply a multi-omics systems medicine approach to characterize uncontrolled pediatric asthma patients and to delineate potential molecular targets for diagnosis and individualized treatment.

The objectives of SysPharmPediA are as follows: (1) to identify non- and/or minimally invasive genetic and beyond-genetic biomarkers and possible relationships/interactions between them that allow classification of different phenotypes of severe uncontrolled pediatric asthma and (2) to construct computational models that effectively predict phenotypes of severe uncontrolled pediatric asthma, using a set of systems-wide biomarkers and their interactions with environmental and clinical factors. In the present work, we aim to introduce and discuss the rationale, design, and cohort description of SysPharmPediA, as well as its strengths, limitations, and challenges that we faced, based on which we will point out some of the possible future research perspectives that such a cohort provides.

## 2. Materials and Methods

### 2.1. Study Design

SysPharmPediA is a multicenter, prospective, observational, pan-European study in a case–control setting. Four tertiary care centers from four European countries (the Netherlands, Germany, Spain, and Slovenia) recruited 145 asthmatic children and adolescents (6–17 years old). All centers obtained approval from their local medical ethics committee (ethics committee of University Regensburg, Germany (18-1034-101); Clinical Research Ethics Committee of the Basque Country, Spain (PI2015075 (SO)); Medical Ethics Committee of the University Medical Center Utrecht (UMC Utrecht), Utrecht, the Netherlands (NL55788.041.15); National Medical Ethics Committee, Slovenia (0120-569/2017/4)), and written informed consents were collected from the parents/caregivers and/or the recruited children when appropriate. This study was retrospectively registered on Clinicaltrials.gov, NCT04865575.

### 2.2. Eligibility Criteria and Main Outcome Definition

Children were eligible to participate when the following criteria were met: (1) aged between 6 and 17 years old, (2) doctor’s diagnosis of asthma, and (3) moderate-to-severe asthmatics under treatment with medication step 3 or higher according to the Global Initiative of Asthma (GINA) guidelines [13]. The recruited children were then classified as presenting with uncontrolled or controlled asthma according to the definition of the SysPharmPediA consortium. Children with uncontrolled asthma were in medication step 3 and had either frequent exacerbations requiring oral corticosteroid (OCS) use (≥1 in the past year) and/or severe exacerbations requiring hospitalization or emergency room (ER) visits in the past year and/or a (childhood) Asthma Control Test (ACT/cACT) [14,15] score ≤19. Patients, who were treated according to GINA step 2, could also be included in the uncontrolled group while being hospitalized due to a severe asthma exacerbation.

Controlled asthmatics were defined by the lack of severe exacerbations requiring OCS use or hospitalizations or ER visits in the past 12 months and an ACT score indicating well-controlled asthma (>19) during the regular asthma check-ups in the past year while being treated with the GINA medication step 3. The workflow diagram for patient recruitment according to the listed inclusion/exclusion criteria is shown in Figure S1. Patients were screened for eligibility using the above-mentioned criteria by the respective recruiting centers. 

### 2.3. Demographics and Clinical Assessment

**Figure 1:**
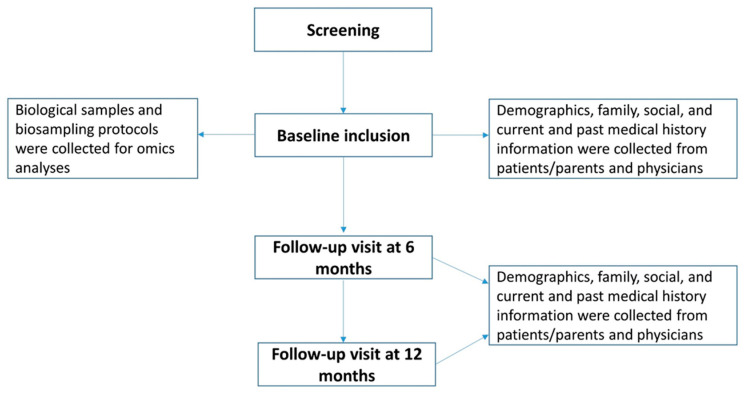
Schematic representation of the study visits schedule. Demographic and current and past medical history assessments were performed at the baseline and follow-up (6 and 12 months) visits. Biosampling protocols (each sample was collected according to a standardized protocol developed for sample tracing, storage, and work-up) and biological specimens were collected from the recruited patients only at the baseline visit.

The scheduled patients’ visits during the SysPharmPediA study and the type of information collected at each study visit are shown in Figure 1. The source of the data collected was a combination of information retrieved from the hospital patients’ files, reported by the treating physicians, and self-reported by patients or parents. In short, this included information on demographics, socioeconomic details, medical history, medication intake, environmental exposures for allergens and smoking, and functional (spirometry) and laboratory assessments (investigation of atopy- and allergy-related disorders). A food diary was requested with registration of intake from 24 h prior to stool collection. Asthma control and quality of life of the patients were assessed using the ACT/cACT [14,15], and the Pediatric Asthma Quality-of-Life Questionnaire (PAQLQ) [16], respectively, and retrieved from the patient’s files. ACT scores range from 5 (poor control of asthma) to 25 (complete control of asthma), with higher scores reflecting greater asthma control [13,14]. An ACT score >19 indicates well-controlled asthma. cACT is suitable for children between 4 and 11 years old, and the ACT for children ≥12 years [13,14]. Medication adherence was assessed using the nine-item Medication Adherence Rating Scale (MARS) [17] and appropriate medication inhalation technique was checked using device-specific inhalation checklists, based on previous research by van der Palen et al. [18].

### 2.4. Atopic Sensitization Assessment

A history of atopic sensitization was derived from patients’ medical records. Patients were defined as atopic if they had sensitization to at least one allergen using skin prick test (SPT) (wheal ≥3 mm) and/or allergen-specific serum immunoglobulin E (IgE) (≥0.35 kUA/L). Allergens tested represent the most common allergens at each respective study center. The list of allergens used for the clinical assessment of atopy is shown in Table S1.

### 2.5. Spirometry Measurements

Spirometry was performed before and after inhalation of 400 µg salbutamol according to the European Respiratory Society/American Thoracic Society (ERS/ATS) guidelines [19], using the following equipment: Viasys Healthcare^®^ MasterScreen Body Jaeger in Spain; Schiller Ganshorn SpiroScout in Slovenia; CareFusion Jaeger^®^ Pneumo Vyntus in the Netherlands; and CareFusion Jaeger^®^ Master Screen Body in Germany. Cooperation and quality of lung function, as well as inhalation technique, were assessed by qualified pulmonary function laboratory personnel.

Predicted percentage and z-score values of lung function measurements (i.e., the forced expiratory volume in the first second (FEV_1_) the forced vital capacity (FVC), and the ratio FEV_1_/FVC) were estimated using the Global Lung Function Initiative (GLI) 2012 equations [20]. The GLI-2012 reference equations are specific for different ethnicities/populations and selecting the most appropriate for non-Caucasian or mixed individuals is uncertain [20]. Thereby, self-reported ethnicity of both parents and genome-wide genetic data were used to select the reference equation that best fit each individual. In case that both parents self-declared different ethnicities, the origin of the children was classified as “Mixed”. Moreover, children were genotyped using the Global Screening Array (GSA, Illumina Inc., San Diego, CA, USA), and principal component analysis (PCA) of genotype data was performed in order to check the accuracy of the self-reported ethnicity and to infer the ethnicity of seven participants with missing self-reported parents’ ethnicity. 

Based on these data and following the ERS recommendations [20], the reference equations used were as follows: (1) “Caucasian group” equation in individuals with European, Latino, North-African ancestry, and mixed ethnicity among these three; (2) “Black group” equation in African individuals; and (3) “Other” equation in the individuals with “Other” or “Mixed” ethnicities, East Asians (who could not be subclassified as North East or South East Asians), and individuals with missing ethnicity and without genotyping data available. To evaluate the adequacy of these reference equations, the distribution of the predicted values was investigated by graphical (i.e., histograms) and statistical methods (i.e., Kolmogorov–Smirnov test). All pre- and post- predicted lung function measurements showed a normal distribution (*p* > 0.05).

### 2.6. Fractional Exhaled Nitric Oxide

The fraction of exhaled nitric oxide (FE_NO_) was measured using Eco Medics CLD 88 sp in Spain; Schiller MS Medisoft FE_NO_ in Slovenia; NIOX VERO^®^ in the Netherlands; and Eco Physics CLD-88 in Germany at a standard flow rate of 50 mL/s according to ERS/ATS guidelines [21]. FE_NO_ values were reported as parts per billion (ppb).

### 2.7. Biosample Collection for Omics Analyses

Biological specimens were obtained from different body compartments for the purposes of specific omics data analyses. Blood, feces, saliva, nasal swabs, and exhaled breath were collected from each participant when possible. To ensure appropriate implementation of research practices, all samples were collected locally and stored under −80 °C according to standard operating procedures (SOPs) harmonized across the consortium centers. Standardized biosampling protocols were developed for samples’ tracing, storage, and work-up for each individual collected sample. All samples (except exhaled breath samples) were transferred to a centralized biobank center (Regensburg, Germany) for storage and subsequent sorting and processing for the respective downstream omics measurements. Collection of exhaled breath was only performed in Spain, Slovenia, and the Netherlands. These samples were stored at 4 °C and subsequently shipped to the Amsterdam UMC, the Netherlands, for breathomics analyses.

### 2.8. Data Collection in STOPPA

In STOPPA, 9–14-year-old children were recruited based on parental reports on asthma in a nationwide twin study [22]. Information on background characteristics, demographics, asthma status, medication, and ACT was collected in questionnaires and obtained from the National Health Registers. At a clinical examination, lung function and capacity (spirometry with reversibility test and exhaled nitric oxide) was performed, and blood (DNA, complete blood count, and plasma aliquots), urine (metabolites), feces (microbiota), and saliva (cortisol) were collected. In total, 13 children fulfilled the eligibility criteria for moderate-to-severe asthma as indicated by the SysPharmPediA study. Ethical approval was obtained from the Ethical Regional Review Board in Stockholm Sweden Dnr 2010/1336-31/3 and 2019-00546.

### 2.9. Platforms of Omics Analyses

Omics analyses (genomics, epigenomics, transcriptomics, proteomics, metabolomics, and microbiome) were carefully selected to provide a comprehensive evaluation of molecular processes in the study participants (Figure S2) guided by published evidence [9]. The omics platforms and technologies used to analyze the different omics layers are summarized in Table S2.

### 2.10. Follow-Up Assessment

Patients were followed-up after 6 and 12 months after the baseline inclusion. Follow-up questionnaires were administered to the participants/parents, doctors, and lung function specialists (Figure 1).

### 2.11. Sample Size Calculation

Data concerning proteome, metabolome, and epigenome data for long-term uncontrolled asthma were lacking in the scientific literature at the beginning of this study; therefore, it was not possible to perform a proper sample size calculation for these omics strategies, based on known effect sizes. For the microbiome analyses, power calculations assuming a small effect size (*φ* > 0.05) to derive the parameters for the Dirichlet-multinomial distribution revealed an excellent power to detect an association (>95%) for a sample size of 100 participants [23]. For that, a fixed conservative number of 10,000 reads per sample (even if 96-indexed runs will yield >20,000 reads per sample) and an alpha of 0.01 were adopted to perform 1000 simulations. For epigenomics, we have enough power to detect differences of 15% in methylation levels for 80% power at a p-value of 1 × 10^−6^ (sample size = 54 cases and 54 controls) [24]. Additionally, it has been shown that epigenome approaches can identify differences between mild and severe asthmatics in studies with small numbers of adult patients (*n* = 10) [25]. Furthermore, preliminary breathomics analysis in the Pharmacogenetics of Asthma Medication in Children: Medication with Anti-inflammatory Effects 2 (PACMAN2) cohort showed statistical differences in exhaled breath profiles between nine long-term uncontrolled asthmatics and seven long-term well-controlled asthmatics [26]. Since the data will be analyzed within the framework of an international multicenter consortium, with the total number of included subjects in this study (*n* = 145), we expect to have sufficient power to detect statistically significant differences between 91 uncontrolled and 54 controlled asthmatics.

### 2.12. Data Management and Statistics

Standardized questionnaires, biosample protocols (SOPs), and clinical assessment documents were designed and maintained by the QNOME online database system (www.qnome.eu (accessed on 01 January 2018)) provided by MaganaMed GmbH, Regensburg, Germany as described previously [27]. Validated master versions in English of the distributed patient’s/parent’s and food diary questionnaires (to collect current and past patients’ history) were designed. They were then locally translated to the four different languages of the recruiting centers. Independent checking was performed by local experts to ensure validated translations. Other questionnaires and documents (e.g., doctor’s questionnaires, lung function, and biosampling protocols) were designed and distributed only in English. After collection of the paper version of the questionnaires, they were digitalized by scanning and subsequent validation, and data entry was then conducted digitally into the QNOME secure server. Data quality checking and independent validation were performed on two separate occasions by the recruiting centers. Expert checks for the data in QNOME were executed at a later stage by independent personnel to ensure data quality and integrity. 

### 2.13. Statistical Analyses

Body mass index (BMI) was converted into z-scores according to the World Health Organization (WHO) growth charts [28]. Data were presented as medians (interquartile ranges—IQRs) or *n* (%) as appropriate. Differences in age, sex, ethnicity, country of inclusion, BMI, and ACT between uncontrolled and controlled asthmatics were evaluated using Mann–Whitney U, and Pearson chi-square tests as appropriate. All statistical comparisons were two-tailed. *p*-values <0.05 were considered significant.

Analyses were performed using R software version 3.6.1 [29] and RStudio Version 1.2.1335 supported by the following packages, dplyr, plyr, tidyr, zscorer, qwraps2, and stats packages.

## 3. Results

**Figure 2:**
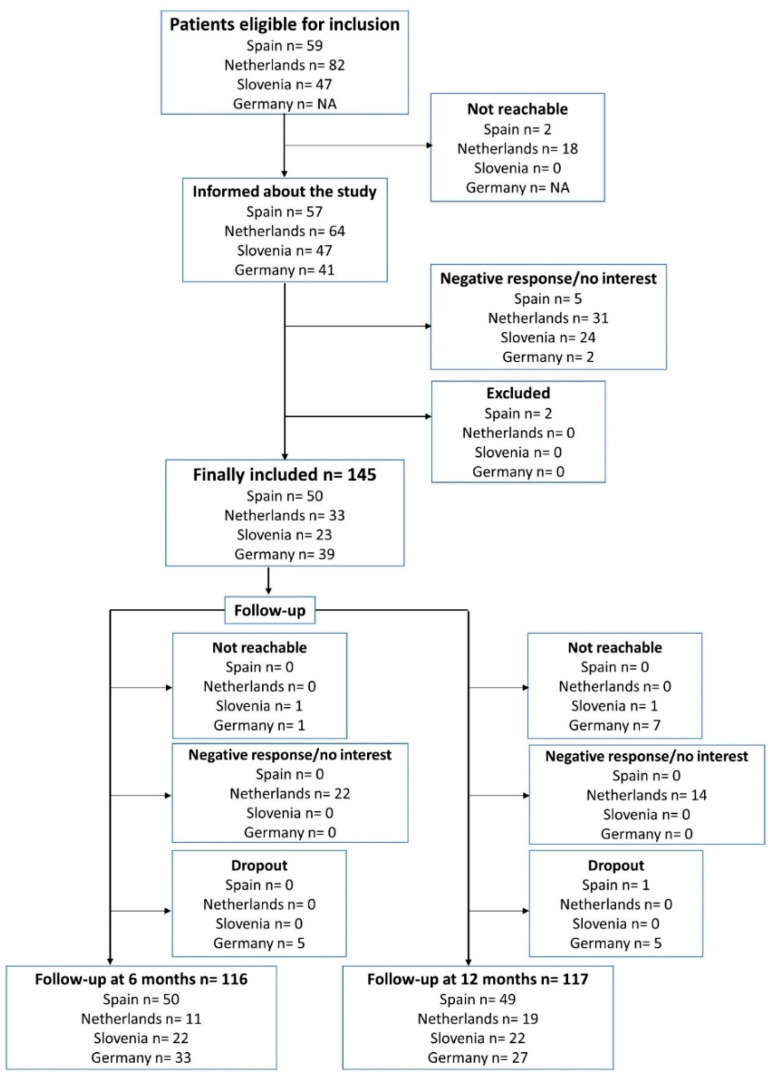
The screening and follow-up phases for the Systems Pharmacology Approach to Uncontrolled Pediatric Asthma (SysPharmPediA) study. Screening details were available from three (Spain, the Netherlands, and Slovenia) out of the four recruiting study centers. NA: not available.

The number of patients screened and deemed eligible for inclusion and follow-ups is depicted in Figure 2. A total of 145 subjects were included in the cohort. 

**Figure 3:**
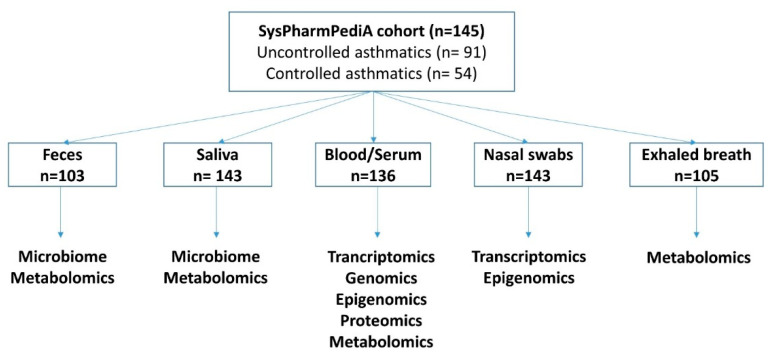
Consort flow diagram showing the total number of recruited subjects and the number of samples collected per body compartment. Five patients provided buccal swabs for genotyping as a replacement of the non-obtained blood samples.

The consort flow diagram for sample collection and omics measurements is shown in Figure 3. The techniques used for omics analyses are summarized in Table S2.

The baseline characteristics for the participants are listed in Table 1. Median age (in years) was 12.0 (IQR = 9.7–14.0) for the recruited uncontrolled asthmatics and 11.7 (IQR = 9.7–13.8) for controlled asthmatics. Forty-three percent of the uncontrolled group and 37% of the controlled group were females. Uncontrolled and controlled asthmatics did not differ regarding age, sex, and BMI z-score (*p*-values >0.05). A higher percentage (93% as compared to 71%) within the asthmatic controls were Caucasian (*p* = 0.006). There was a statistically significant difference in the percentages of uncontrolled and controlled asthmatics within different countries of inclusion (*p* = 0.014). Atopy was present in 89% of uncontrolled asthmatics and 85% in controlled asthmatics. Uncontrolled asthmatics had lower median values for ACT (*p* < 0.001).

The median age of the STOPPA participants was 11.8 years and 30.8% were females. Of the total sample, positive Phadiatop as a marker of atopic sensitization was reported among 75% (Table S3).

## 4. Discussion

The SysPharmPediA study demonstrates a pan-European attempt to molecularly and clinically characterize pediatric patients with uncontrolled asthma, using a multi-omics approach. Portraying diagnostic and therapeutic clinical decisions based merely on the symptomatic clinical profile may not be sufficient to adequately control asthma. Hence, we aim to integrate the omics information from different layers (levels), using a systems medicine approach to accurately assess the pathophysiological pathways underlying uncontrolled pediatric asthma and to elucidate targets for individualized diagnosis and treatment. 

In the SysPharmPediA consortium, collaboration between multiple clinical centers and research groups within four pan-European countries allows intra- and interdisciplinary interactions, which is likely to help in the understanding of the complex pathophysiological processes of uncontrolled childhood asthma. All consortium partners worked in this study under a priori unified objectives and used a harmonized work frame, implementing standardized protocol of patients’ recruitment and clinical assessment, SOPs, and a centralized biobank facility for sample collection, storage, and work-up, which ensured proper implementation of research practices and optimum methodological conduct.

Adoption of novel omics technologies allowing analyses from different non- and minimally invasive sampling compartments in this study, coupled with the future utilization of advanced computational and systems biology approaches, will allow the molecular mapping of hundreds of biomolecules with the potential to reveal noninvasive biomarkers for personalized diagnosis and/or treatment of uncontrolled asthma. Recent advances in statistical and computational techniques of multi-omics layers-based integration [9,30] allow a comprehensive overview of how different biomolecules interact in the pathophysiology of the disease processes and could also support and improve molecular classification of patients compared to single-omics layers [31]. Thus, such an approach could provide refined phenotyping and/or endotyping of the patients [9].

SysPharmPediA has a number of important advantages/strengths. First, the low prevalence of childhood moderate-to-severe and uncontrolled asthma [5,32], necessitates the collaboration of multiple centers from different countries to allow recruitment of sufficient sample size for adequate molecular and clinical characterization. The multicenter nature of this study allowed the recruitment of a relatively large number of patients with a phenotype scarcely analyzed in pediatric studies of asthma—uncontrolled moderate-to-severe asthma—and, therefore, provides more reliable findings than single-center or single-country studies. In addition, the sample size of the recruited children is considered high compared to other multi-omics studies investigating the childhood severe asthma phenotype, such as the U-BIOPRED and SARP [10,11,12]. Second, the multi-omics approach utilized here allows comprehensive mapping of hundreds of noninvasive biomolecules and might provide a better understanding of the asthma-specific molecular signatures in comparison with the single-omics studies, which are nowadays widely available. It is believed that the more omics layers we integrate, the higher power could be achieved, and hence, a smaller sample size will be required to adequately classify patients based on their molecular profiles [31]. Third, the patients were followed-up 6 and 12 months after the baseline visit, which might help to assess longitudinal variations in the disease-specific processes. Finally, the integrated harmonized framework based on the uniform standardized protocols, SOPs, and centralized biobank helped to ensure the application of unified research methodology across the different study centers, and hence increased data quality by limiting any methodological or technical variations and will offer an optimum platform for collaboration/validation with other multi-omics asthma studies.

However, this study has also some limitations. A large percentage of the included children were white Caucasian (79%), hence the studied population might not be representative of other ethnicities. Second, we have only followed-up children over a relatively short time (at 6 and 12 months after baseline inclusion), which might not be sufficient to adequately capture time-dependent pathophysiological changes occurring over longer periods. However, looking at the retrospective history of these patients (e.g., asthma symptoms/exacerbation history in the past 12 months prior to inclusion) may help us to get in-depth information on a longer period of time. In addition, biological samples were collected only at the baseline visit and not at the 6- and 12-month follow-up visits. This was mainly related to limited resources within the project, and the burden that would have been caused to the patients resulting from the potential collection of several additional biological samples at the follow-up visits. Third, differences between countries (such as dietary habits, environmental exposures, genetic background, and healthcare for asthmatics) and the distribution of uncontrolled and controlled asthmatics within the countries of inclusion might additionally complicate the analyses. However, we ensured that both uncontrolled and controlled asthmatics were included in each center of inclusion to reduce potential bias related to the inclusion center. Finally, loss to follow-up of patients (attrition bias) is a common issue in longitudinal cohort studies [33], which, in turn, might lead to some biased estimates in the assessment of time-dependent omics and clinical variations in our SysPharmPediA cohort. 

In addition to the strengths and limitations discussed, we had to overcome some challenges in order to successfully include the patients at all recruiting sites. First, the patient’s/parent’s questionnaires were translated to the native language of each recruiting country to ensure that there are no misinterpretations by the children or the parents of the questions or information described in these questionnaires. Furthermore, we had to describe the asthma medications reported/utilized by the patients by the local trade names for ease of recognition, which was challenging as they differ in each county. However, the usage of a centralized database system for the questionnaires, QNOME [27], made it feasible to unify the information collected despite the differences between countries in languages or medication usage. Second, variations in the legislation between different centers/countries required each recruiting center to obtain ethical approval from the respective local medical ethical committees for the biosampling and the analyses. Although all centers managed to receive all necessary approvals for most types of biosamples collected, it was not feasible to collect exhaled breath samples from the German patients, because the collection device did not meet the German legislation requirements. Differences in legislation and ethical approval requirements needed for each study center hinder the collaborative efforts and the multi-omics projects. In addition, the current strict legislations for data/samples privacy and ownership further hinder the multicenter collaborative efforts. The EU General Data Protection Regulation (GDPR) attempts to regulate and harmonize data privacy across Europe [34]. However, this might influence data sharing and centralized biobanking [35]. 

Although the biosampling process was minimally invasive, some children and/or parents were not comfortable providing multiple samples from various (body) compartments simultaneously, which slowed the recruitment process. Therefore, we extended the time for the recruitment phase, to reach the required sample size. Moreover, some of the children/adolescents felt uncomfortable and/or refused to provide some types of samples, such as feces, which resulted in a decrease of the total numbers of this sample type as compared to the others (saliva, blood, or nasal swabs). Interestingly, this differed between study centers. For example, the number of patients who were unwilling to provide stool samples was higher in the Netherlands compared to that in Spain. This might be related to specific cultural differences in the respective countries of inclusion. We tried to overcome this obstacle by offering to visit and collect these samples from the patients’ homes whenever appropriate. 

In general, multi-omics projects in asthma are scarce, particularly for children. Therefore, SysPharmPediA offers a unique multi-omics cohort with uncontrolled and controlled pediatric asthma patients and will provide an opportunity to further understand childhood asthma development. Ongoing and future collaboration with other multi-omics projects studying childhood asthma, such as the U-BIOPRED [10] and the Swedish Twin Study on Perinatal Characteristics to Prevent Asthma (STOPPA) [22], could achieve (better) reliability of findings applicable for various ethnic groups, as well as further enhance multi-omics asthma research, that is so much needed for future application of omics in clinical practice and precision medicine.

Based on our experience, there are conclusions that we can draw from the SysPharmPediA consortium, at this point (summarized in Box 1). This knowledge might guide other researchers, who are willing to conduct multi-omics studies, to tackle specific molecular signatures of various diseases.

In summary, SysPharmPediA will provide comprehensive directions to study disease development in children with uncontrolled pediatric asthma using a noninvasive multi-omics approach. This also provides potential to reveal noninvasive biomarkers, which could be used for personalized diagnosis and/or treatment, and may reveal mechanistic disease pathways using a systems-wide medicine approach.

Box 1 **Knowledge acquired from the SysPharmPediA consortium.**

1. The establishment of a multicenter multi-omics project in children with severe uncontrolled asthma has large personnel and computational facility requirements.
2. Advances in high-throughput omics technologies (and the relatively low costs of omics analyses nowadays), allow multi-omics projects to become more and more feasible compared with the last decade.
3. The relatively small number of children with severe asthma (2–10% of the total childhood asthma population) makes the recruitment of such a unique cohort quite a challenging task and requires collaboration between multiple study centers from multiple countries.
4. Differences in medical/ethical legislations and regulations in different European countries make study preparations difficult and often compromise study uniformity.
5. Obtaining ethical approval in several different European countries is time consuming and, therefore, enough time should be scheduled for this in advance.
6. The recruitment can be hindered by the unwillingness of study participants and/or their guardians to provide multiple samples from multiple body compartments and/or by being uncomfortable to provide specific types of samples (e.g., feces and blood).
7. Using standardized operating procedures (SOPs) and protocols guarantees the uniform methodological conduct and appropriate handling, storage, and processing of samples.
8. Working under a harmonized work frame and using a validated database system to handle questionnaires and documents in different languages is important to ensure delivery of the study objectives.
9. Using a centralized biobank is necessary to unify methodological conducts and omics measurements.
10. Recent European regulations, such as the EU General Data Protection Regulation (GDPR), might influence decisions/actions related to omics and data sharing between countries and study centers.
11. Differences in the origin (nationality and/or ethnicity) of the recruited participants are favorable to reach more generalizable findings and/or conclusions from the study. However, this can also come at the expense of higher variability in confounding factors, such as different environments of exposure, dietary habits, and genetic background of the recruited subjects.
12. A project coordinator who routinely organizes monthly/bi-monthly online meetings with all study partners to discuss current status, study progress, and how to overcome challenges in a timely manner is crucial.
13. Aside from principal investigators within a project, it is important to also involve young researchers with hands-on practical experience within each and every team, who can provide input on the content of the study, apart from executing practical tasks related to the project.
14. Yearly face-to-face meetings at the different study locations (each time hosted by another partner within the consortium) are essential for the good collaboration within such an international project. This allows all cooperating partners within the project to witness directly how the setting of the respective clinical study center is being adapted for the needs of the particular project.

## Figures and Tables

**Table 1:** Baseline characteristics for the study participants.

Characteristics (SysPharmPediA)	All Recruited Subjects (*n* = 145)	Uncontrolled Asthmatics (*n* = 91)	Controlled Asthmatics (*n* = 54)
Age in years, median (IQR)	11.93 (9.65, 14.00)	12.00 (9.68, 14.00)	11.74 (9.65, 13.84)
Female, *n* (%)	59/145 (41%)	39/91 (43%)	20/54 (37%)
Ethnicity, *n* (%) #			
Caucasian	115/145 (79%)	65/91 (71%)	50/54 (93%)
Latino	10/145 (7%)	7/91 (8%)	3/54 (6%)
African	6/145 (4%)	6/91 (7%)	0/54 (0%)
Asian	2/145 (1%)	1/91 (1%)	1/54 (2%)
Mixed/Other	12/145 (8%)	12/91 (13%)	0/54 (0%)
BMI z-score, median (IQR)	0.57 (−0.35, 1.38) (*n* = 144)	0.50 (−0.30, 1.43) (*n* = 90)	0.74 (−0.45, 1.27) (*n* = 54)
Cesarean section, *n* (%)	27/139 (19%)	15/86 (17%)	12/53 (23%)
Breast feeding, duration in months, median (IQR)	4.00 (0.00, 9.00) (*n* = 137)	5.50 (0.00, 9.00) (*n* = 86)	4.00 (1.00, 9.00) (*n* = 51)
Current living environment, *n* (%) ##			
City	58/140 (41%)	43/87 (49%)	15/53 (28%)
City center	11/140 (8%)	8/87 (9%)	3/53 (6%)
Rural area	18/140 (13%)	11/87 (13%)	7/53 (13%)
Village	53/140 (38%)	25/87 (29%)	28/53 (53%)
Active and/or passive smoking exposure during pregnancy, *n* (%)	35/130 (27%)	20/79 (25%)	15/51 (29%)
Current active/passive smoking, *n* (%)	42/138 (30%)	26/87 (30%)	16/51 (31%)
Country of inclusion, *n* (%)			
Spain	50/145 (34%)	35/91 (38%)	15/54 (28%)
Germany	39/145 (27%)	20/91 (22%)	19/54 (35%)
The Netherlands	33/145 (23%)	26/91 (29%)	7/54 (13%)
Slovenia	23/145 (16%)	10/91 (11%)	13/54 (24%)
Atopy, *n* (%)	121/138 (88%)	76/85 (89%)	45/53 (85%)
Diagnosed allergic rhinitis (ever), *n* (%)	101/137 (74%)	62/85 (73%)	39/52 (75%)
Diagnosed allergic conjunctivitis (ever), *n* (%)	87/134 (65%)	55/83 (66%)	32/51 (63%)
Asthma Control Test (ACT), median (IQR)	23.00 (19.00, 25.00) (*n* = 140)	20.00 (17.00, 23.00) (*n* = 88)	24.50 (23.00, 25.00) (*n* = 52)
Current asthma medication intake §, *n* (%)			
ICS	145/145 (100%)	91/91 (100%)	54/54 (100%)
SABA §§	133/142 (94%)	85/89 (96%)	48/53 (91%)
LABA	134/143 (94%)	83/90 (92%)	51/53 (96%)
OCS	31/137 (23%)	30/84 (36%)	1/53 (2%)
LTRA	25/131 (19%)	18/84 (21%)	7/47 (15%)
Omalizumab	14/139 (10%)	13/86 (15%)	1/53 (2%)
Mepolizumab	2/137 (1%)	2/85 (2%)	0/52 (0%)
Spirometry % predicted, median (IQR)			
FEV_1_ pre-salbutamol	94.01 (82.74, 102.96) (*n* = 142)	95.20 (82.05, 102.18) (*n* = 89)	92.64 (86.08, 103.26) (*n* = 53)
FEV_1_ post-salbutamol	99.40 (89.97, 108.76) (*n* = 140)	99.66 (91.90, 108.01) (*n* = 88)	97.63 (89.44, 109.35) (*n* = 52)
FEV_1_/FVC pre-salbutamol	95.42 (87.20, 100.38) (*n* = 142)	94.05 (85.85, 98.99) (*n* = 89)	97.25 (89.24, 102.76) (*n* = 53)
FEV_1_/FVC post-salbutamol	99.31 (92.35, 103.56) (*n* = 140)	98.79 (90.58, 103.69) (*n* = 89)	99.86 (94.64, 103.51) (*n* = 52)
Spirometry z-score, median (IQR)			
FEV_1_ pre-salbutamol	−0.49 (−1.44, 0.26) (*n* = 142)	−0.40 (−1.55, 0.20) (*n* = 89)	−0.63 (−1.18, 0.28) (*n* = 53)
FEV_1_ post-salbutamol	−0.05 (−0.84, 0.73) (*n* = 140)	−0.03 (−0.68, 0.69) (*n* = 88)	−0.20 (−0.90, 0.75) (*n* = 52)
FEV_1_/FVC pre-salbutamol	−0.68 (−1.65, 0.06) (*n* = 142)	−0.89 (−1.84, −0.15) (*n* = 89)	−0.42 (−1.52, 0.41) (*n* = 53)
FEV_1_/FVC post-salbutamol	−0.10 (−1.05, 0.56) (*n* = 140)	−0.18 (−1.28, 0.59) (*n* = 88)	−0.02 (−0.77, 0.55) (*n* = 52)
FE_NO_ (ppb), median (IQR)	16.35 (8.88, 41.25) (*n* = 124)	21.70 (11.00, 50.38) (*n* = 80)	10.50 (6.53, 17.18) (*n* = 44)
Whole-blood cellular counts (absolute count × 10^9^/L), median (IQR)			
Eosinophils	0.37 (0.21, 0.62) (*n* = 126)	0.46 (0.23, 0.74) (*n* = 80)	0.35 (0.18, 0.45) (*n* = 46)
Neutrophils	3.27 (2.43, 4.24) (*n* = 126)	3.31 (2.44, 4.30) (*n* = 80)	3.20 (2.40, 4.04) (*n* = 46)
Lymphocytes	2.58 (2.23, 3.10) (*n* = 126)	2.71 (2.23, 3.24) (*n* = 80)	2.51 (2.21, 2.90) (*n* = 46)
Basophils	0.04 (0.03, 0.06) (*n* = 126)	0.04 (0.03, 0.06) (*n* = 80)	0.04 (0.02, 0.06) (*n* = 46)
Monocytes	0.53 (0.41, 0.66) (*n* = 126)	0.53 (0.41, 0.66) (*n* = 80)	0.54 (0.43, 0.65) (*n* = 46)
Leucocytes	7.16 (5.79, 8.40) (*n* = 128)	7.30 (5.95, 9.09) (*n* = 82)	6.99 (5.71, 7.60) (*n* = 46)
Erythrocytes	4.97 (4.64, 5.48) (*n* = 126)	5.04 (4.60, 7.58) (*n* = 80)	4.83 (4.69, 5.02) (*n* = 46)
Thrombocytes	278.00 (250.50, 338.50) (*n* = 127)	277.00 (253.00, 349.00) (*n* = 81)	278.50 (243.75, 324.50) (*n* = 46)

Categorical variables are described as *n* (% of *n*), and continuous variables as median (interquartile range, (IQR)). ACT: Asthma Control Test; scores range from 5 (poor control of asthma) to 25 (complete control of asthma), with higher scores reflecting greater asthma control. An ACT score >19 indicates well-controlled asthma. BMI: body mass index, FEV_1_: forced expiratory volume in 1 s, FVC: forced vital capacity, FE_NO_: fraction of exhaled nitric oxide, ICS: inhaled corticosteroids, LTRA: leukotriene antagonist, SABA: short-acting beta agonist, LABA: long-acting beta agonist, OCS: oral corticosteroids. # Seven subjects did not recode their ethnicity, and for six of them with genotyping data available, it was estimated from a principal component analysis (PCA). ## Five patients double recorded living in both rural area and village, and they were assigned as living in a village. § Medication intake based on the physician-reported use of medication in the last 12 months. §§ SABA usage includes those who were regular/current users of SABA through inhalers and/or nebulizers.

## Data Availability

For clinical and other omics data generated within the SysPharmPediA study, the authors will make these data available upon specific requests subject to the requestor obtaining ethical, research, data access, and collaboration approvals from the SysPharmPediA study management board. Requests can be sent to a.h.maitland@amsterdamumc.nl.

## Supplementary Materials

The following are available online at https://www.mdpi.com/article/10.3390/jpm11060484/s1, Figure S1: The inclusion/exclusion criteria and the main outcome definition in the SysPharmPediA cohort, Figure S2: Different omics blocks (layers) measured in the SysPharmPediA cohort. Table S1: List of the aero- and food-allergens tested by the skin prick test (SPT) and/or specific IgE for diagnosis of atopy in the recruited subjects, Table S2: Summary of different omics platforms and technologies used in the SysPharmPediA study, Table S3: Baseline characteristics for the STOPPA study participants that follow the eligibility criteria by the SysPharmPediA study.
