# Supplementary material for: A System Pharmacology Multi-Omics Approach toward Uncontrolled Pediatric Asthma
Source: J Pers Med. 2021 May 28;11(6):484. doi: 10.3390/jpm11060484 (PMC8227234; doi:10.3390/jpm11060484)
Supplement: Supplementary file 1 [file jpm-11-00484-s001.zip › Figure S2.pdf]

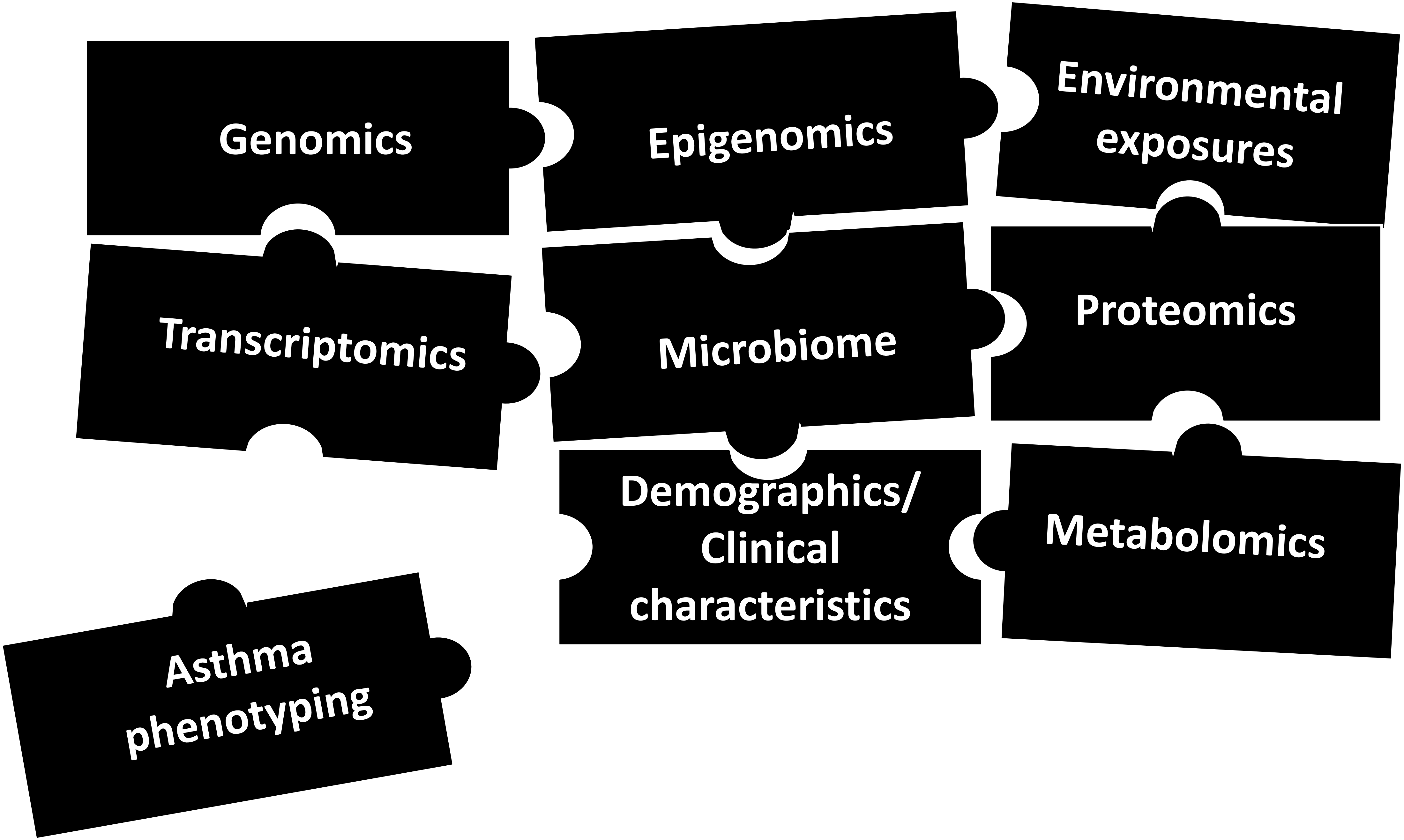
Genomics
Epigenomics
Environmental exposures
Transcriptomics
Microbiome
Proteomics
Demographics/ Clinical characteristics
Metabolomics
Asthma phenotyping
