# Supplementary material for: A System Pharmacology Multi-Omics Approach toward Uncontrolled Pediatric Asthma
Source: J Pers Med. 2021 May 28;11(6):484. doi: 10.3390/jpm11060484 (PMC8227234; doi:10.3390/jpm11060484)
Supplement: Supplementary file 1 [file jpm-11-00484-s001.zip › Supplementary material.pdf]

A system pharmacology multi-omics approach towards uncontrolled pediatric asthma

Mahmoud I. Abdel-Aziz [1,2,†], Anne H. Neerincx [1,†], Susanne J. H. Vijverberg [1], Simone Hashimoto [1,3], Paul Brinkman [1], Mario Gorenjak [4], Antoaneta A. Toncheva [5], Susanne Harner [5], Susanne Brandstetter [6], Christine Wolff [6], Javier Perez-Garcia [7], Anna M. Hedman [8], Catarina Almqvist [8,9], Paula Corcuera-Elosegui [10], Javier Korta-Murua [10,11], Olaia Sardón-Prado [10,11], Maria Pino-Yanes [7,12,13], Uroš Potočnik [4,14], Michael Kabesch [5,6], Aletta D. Kraneveld [15], Anke H. Maitland-van der Zee [1,3,*] and on behalf of the SysPharmPediA consortium

1 Department of Respiratory Medicine, Amsterdam UMC, University of Amsterdam, 1105 AZ Amsterdam, The Netherlands; m.i.ibrahim@amsterdamumc.nl (M.I.A.-A.); a.vanstuyvenbergneerincx@amsterdamumc.nl (A.H.N.); s.j.vijverberg@amsterdamumc.nl (S.J.H.V.); s.hashimoto@amsterdamumc.nl (S.H.); p.brinkman@amsterdamumc.nl (P.B.)

2 Department of Clinical Pharmacy, Faculty of Pharmacy, Assiut University, 71526 Assiut, Egypt

3 Department of Pediatric Respiratory Medicine, Emma Children's Hospital, Amsterdam UMC, 1105 AZ Amsterdam, The Netherlands

4 Center for Human Molecular Genetics and Pharmacogenomics, Faculty of Medicine, University of Maribor, 2000 Maribor, Slovenia; mario.gorenjak@um.si (M.G.); uros.potocnik@um.si (U.P.)

5 Department of Pediatric Pneumology and Allergy, University Children's Hospital Regensburg (KUNO) at the Hospital St. Hedwig of the Order of St. John, University of Regensburg, D-93049 Regensburg, Germany; Antoaneta.Toncheva@klinik.uni-regensburg.de (A.A.T.); Susanne.Harner@barmherzige-regensburg.de (S.H.); Michael.Kabesch@barmherzige-regensburg.de (M.K.)

6 Science and Development Campus Regensburg (WECARE), University Children's Hospital Regensburg (KUNO) at the Hospital St. Hedwig of the Order of St. John, University of Regensburg, D-93049 Regensburg, Germany; Susanne.Brandstetter@barmherzige-regensburg.de (S.B.); Christine.Wolff@barmherzige-regensburg.de (C.W.)

7 Genomics and Health Group, Department of Biochemistry, Microbiology, Cell Biology and Genetics, Universidad de La Laguna, 38200 San Cristóbal de La Laguna, Santa Cruz de Tenerife, Spain; jpegarci@ull.edu.es (J.P.-CG.); mdelpino@ull.edu.es (M.P.-Y.)

8 Department of Medical Epidemiology and Biostatistics, Karolinska Institutet, 171 77 Stockholm, Sweden; anna.hedman@ki.se (A.M.H.); Catarina.Almqvist@ki.se (C.A.)

9 Pediatric Allergy and Pulmonology Unit, Astrid Lindgren Children's Hospital, Karolinska University Hospital, 171 77 Stockholm, Sweden

10 Division of Pediatric Respiratory Medicine, Hospital Universitario Donostia, 20014 San Sebastián, Spain; PAULA.CORCUERAELOSEGUI@osakidetza.eus (C.P.E.); JOSEJAVIER.KORTAMURUA@osakidetza.eus (C.K.M.); OLAIA.SARDONPRADO@osakidetza.eus (O.S.P.)

11 Department of Pediatrics, University of the Basque Country (UPV/EHU), 20014 San Sebastián, Spain

12 CIBER de Enfermedades Respiratorias, Instituto de Salud Carlos III, 28029 Madrid, Comunidad de Madrid, Spain

13 Instituto de Tecnologías Biomédicas (ITB), Universidad de La Laguna, 38200 San Cristóbal de La Laguna, Santa Cruz de Tenerife, Spain

14 Laboratory for Biochemistry, Molecular Biology and Genomics, Faculty of Chemistry and Chemical Engineering, University of Maribor, 2000 Maribor, Slovenia

15 Division of Pharmacology, Utrecht Institute for Pharmaceutical Sciences, Faculty of Science, Utrecht University, 3584CG Utrecht, The Netherlands; A.D.Kraneveld@uu.nl

* Correspondence: a.h.maitland@amsterdamumc.nl

† Shared first co-authorship.

Supplementary Materials

Figures:

Figure S1: The inclusion/exclusion criteria and the main outcome definition in the SysPharmPediA cohort
Figure S2: Different omics blocks (layers) measured in the SysPharmPediA cohort

Tables:

Table S1: List of the aero- and food-allergens tested by the skin prick test (SPT) and/or specific IgE for diagnosis of atopy in the recruited subjects
Table S2: Summary of different omics platforms and technologies used in the SysPharmPediA study
Table S3: Baseline characteristics for the STOPPA study participants that follow the eligibility criteria by the SysPharmPediA study

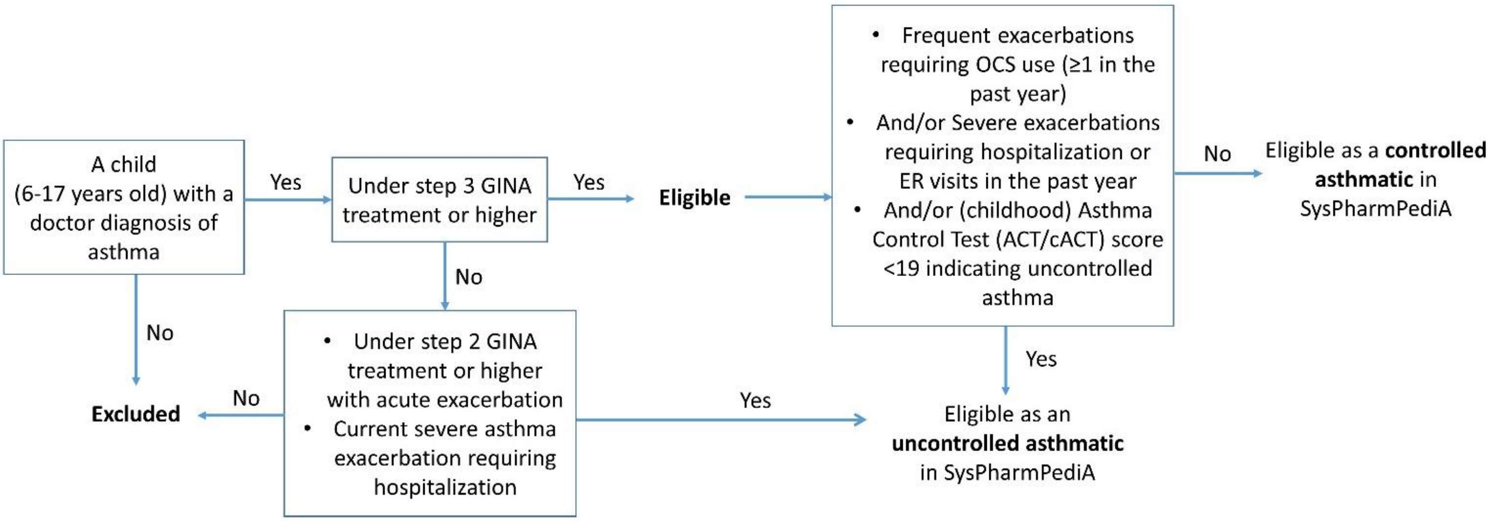

Figure S1: The inclusion/exclusion criteria and the main outcome definition in the SysPharmPediA cohort.

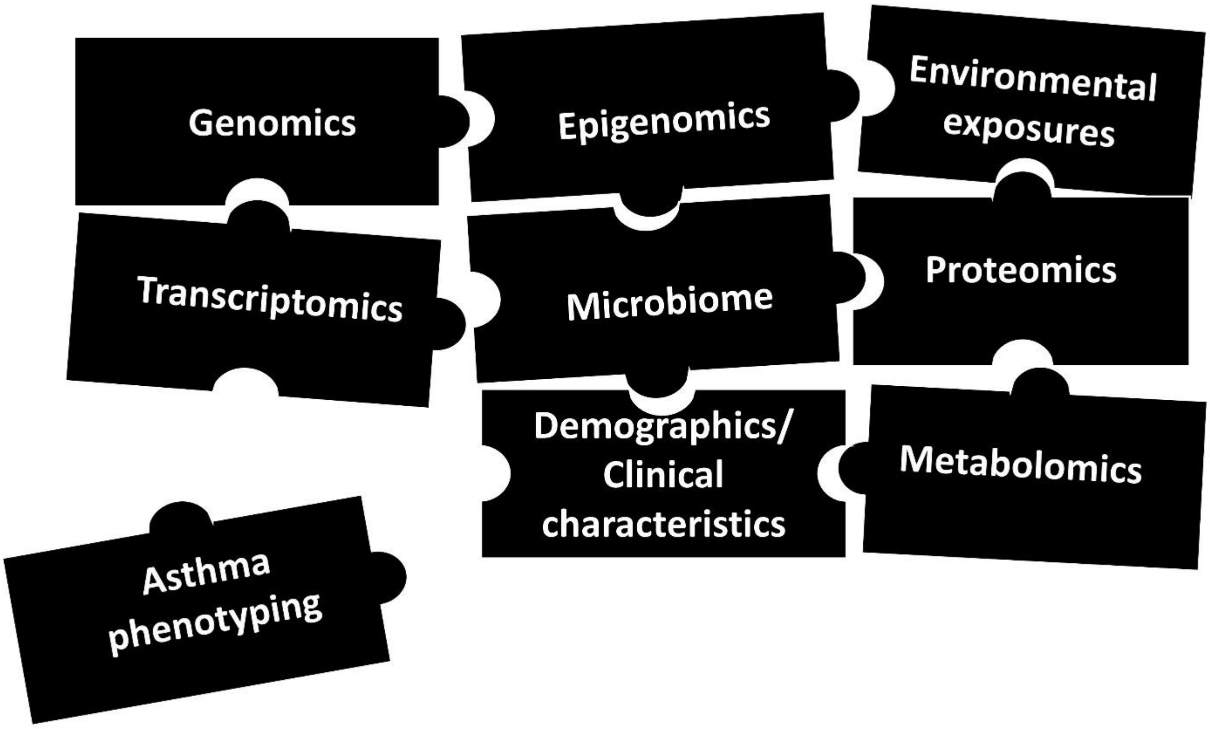

Figure S2: Different omics blocks (layers) measured in the SysPharmPediA cohort. Each omics block, together with demographic details, clinical assessment, and history of environmental exposures and dietary intake, may provide part of the information (puzzle piece) that will help us to phenotype, and to better characterize (molecularly and clinically) uncontrolled and controlled asthmatics within the SysPharmPediA study. Integrating information from multiple puzzle pieces could help to provide an in-depth picture of the pathophysiological processes underlying uncontrolled childhood asthma and further refine identified phenotypes.

Table S1: List of the aero- and food-allergens tested by the skin prick test (SPT) and/or specific IgE for diagnosis of atopy in the recruited subjects.

1. Birch<br>2. Alder<br>3. Hazel<br>4. Ash<br>5. Grass mixture<br>6. Rye<br>7. Mugwort (*Artemisia vulgaris*)<br>8. Ragweed (Ambrosia)<br>9. Buckhorn (*Plantago lanceolata*)<br>10. *Olea europea*<br>11. House dust mite (*Dermatophagoides pteronyssinus*)<br>12. House dust mite (*Dermatophagoides farina*)<br>13. *Alteraria alternata*<br>14. *Cladosporium herbarum*<br>15. *Aspergillus fumigatus*<br>16. *Penicillium notatum*<br>17. Cat (Feline epithelia)<br>18. Dog (Canine epithelia)	19. Egg<br>20. Nut<br>21. Peanut<br>22. Hazelnut<br>23. Pistachio<br>24. Almond<br>25. Soy<br>26. Milk<br>27. Fish<br>28. Lentils<br>29. Beans<br>30. Chickpea<br>31. Others reported by the physician.

Table S2: Summary of different omics platforms and technologies used in the SysPharmPediA study.

Omics layers	Technique and platform used
Genomics	DNA samples (peripheral whole blood, buccal swabs and in some subjects nasal swabs) were subjected to genotyping using the Infinium Global Screening Array-24 v2.0 and v3.0 Kits according to the manufacturer instructions. The BeadChip assures coverage of > 654,000 single nucleotide polymorphisms (SNPs).
Epigenomics	Methylation patterns are evaluated in peripheral whole blood and nasal swabs using the Infinium MethylationEPIC Kit, which provides the option to study more than 850,000 methylation sites (CpG islands, genes, enhancers, DNase hypersensitive sites). As little as 450 ng of DNA was sufficient to perform the bisulfite conversion and then load the samples on the EPIC BeadChip.
Transcriptomics	RNA obtained from peripheral whole blood using the PAXgene tubes are subjected to transcriptomic analyses based on the single-cell tagged reverse transcription (STRT) RNAseq method.
Microbiome	Total DNA was extracted from SysPharmPediA saliva and stool samples using the QIAamp DNA Mini Kit (Qiagen) run on the QIAcube semi-automated. Samples are PCR amplified for 16S ribosomal RNA (rRNA) using specified primers targeting V1-V2 and V3-V4 regions and sequencing performed using the MiSeq v3 platform according to the Illumina protocol.
Metabolomics	Serum and stool samples from the SysPharmPediA study are analyzed using the MxP Quant 500 Kit (Biocrates Life Sciences AG, Innsbruck, Austria) and liquid chromatography-electrospray ionization-tandem mass spectrometric (LC-ESI-MS/MS, shortly: LC-MS/MS) and flow injection-electrospray ionization-tandem mass spectrometric (FIA-ESI-MS/MS) measurements performed. The assay allows determination of more than 600 substances comprising of a broad range of small molecules (e.g. amino acids and amino acids related, biogenic amines, carboxylic acids) and a large set of lipids (e.g. acylcarnitines, ceramides, diacylglycerides, triglycerides).
Exhaled breath metabolomics	For volatile metabolites, exhaled breath samples were collected on thermal desorption tubes and are analysed through thermal desorption (TD100; Markes) coupled to gas chromatography-mass spectrometry (Shimadzu QP2010, Kyoto, Japan). The R package xcms is used to align the obtained samples. Fragment ions of interest is manually checked in the raw chromatograms, and corresponding metabolites are tentatively identified using National Institute of Standards and Technology library (NIST, Gaithersburg, MD).
Proteomics (multiplex)	Serum samples from the SysPharmPediA study are analysed using Luminex assay to measure a targeted panel of cytokines and chemokines linked to inflammation.

Table S3: Baseline characteristics for the STOPPA study participants that follow the eligibility criteria by the SysPharmPediA study.

Characteristics (STOPPA)	All recruited subjects (n=13)	Uncontrolled asthmatics (n=7)	Controlled asthmatics (n=6)
**Age in years, median (IQR)**	11.8 (10.8, 13.0)	11.2 (10.5, 12.1)	12.6 (11.8, 13.5)
**Female, n (%)**	4/13 (30.8%)	4/7 (57.1%)	0/6 (0%)
**Atopy (Phadiatop), n (%)**	9/12 (75%)	3/6 (50%)	6/6 (100%)

Categorical variables are described as n (% of n), and continuous variables as median (interquartile range, (IQR)).
