# Supplementary material for: A System Pharmacology Multi-Omics Approach toward Uncontrolled Pediatric Asthma
Source: J Pers Med. 2021 May 28;11(6):484. doi: 10.3390/jpm11060484 (PMC8227234; doi:10.3390/jpm11060484)
Supplement: Supplementary file 1 [file jpm-11-00484-s001.zip › SysPharmPediA consortium list.pdf]

SysPharmPediA consortium list

The SysPharmPediA consortium wishes to acknowledge the help and expertise of the following individuals and groups without whom, the study would not have been possible.

SysPharmPediA members:

**Name:**	**Country:**	**Affiliation:**
Olaia Sardón-Prado MD, PhD	Spain	Division of Pediatric Respiratory Medicine, Hospital Universitario Donostia, San Sebastián, Spain<br><br>Department of Pediatrics, University of the Basque Country (UPV/EHU), San Sebastián, Spain.
Paula Corcuera-Elósegui MD, phD	Spain	Division of Pediatric Respiratory Medicine, Hospital Universitario Donostia, San Sebastián, Spain.
Javier Korta-Murua MD, PhD	Spain	Division of Pediatric Respiratory Medicine, Hospital Universitario Donostia, San Sebastián, Spain<br><br>Department of Pediatrics, University of the Basque Country (UPV/EHU), San Sebastián, Spain.
Mahmoud I. Abdel-Aziz MSc	The Netherlands	Department of Respiratory Medicine, Amsterdam UMC, University of Amsterdam, Amsterdam, The Netherlands.<br><br>Department of Clinical Pharmacy, Faculty of Pharmacy, Assiut University, Assiut, Egypt.
Paul Brinkman MSc	The Netherlands	Department of Respiratory Medicine, Amsterdam UMC, University of Amsterdam, Amsterdam, The Netherlands.
Susanne J. H. Vijverberg PhD	The Netherlands	Department of Respiratory Medicine, Amsterdam UMC, University of Amsterdam, Amsterdam, The Netherlands.
Anne H. Neerincx PhD	The Netherlands	Department of Respiratory Medicine, Amsterdam UMC, University of Amsterdam, Amsterdam, The Netherlands.
Simone Hashimoto MD PhD	The Netherlands	Department of Respiratory Medicine, Amsterdam UMC, University of Amsterdam, Amsterdam, The Netherlands.<br><br>Department of Pediatric Respiratory Medicine, Emma Children's Hospital, Amsterdam UMC, Amsterdam, The Netherlands.
Aletta D. Kraneveld PhD	The Netherlands	Division of Pharmacology, Utrecht Institute for Pharmaceutical Sciences, Faculty of Science, Utrecht University, Utrecht, the Netherlands.

		Institute for Risk Assessment Sciences, Faculty of Veterinary Medicine, Utrecht University, Utrecht, the Netherlands.
Anke H. Maitland-van der Zee PharmD, PhD	The Netherlands	Department of Respiratory Medicine, Amsterdam UMC, University of Amsterdam, Amsterdam, The Netherlands.<br><br>Department of Pediatric Respiratory Medicine, Emma Children's Hospital, Amsterdam UMC, Amsterdam, The Netherlands.
Maria Pino-Yanes, PhD	Spain - Tenerife	Genomics and Health Group, Department of Biochemistry, Microbiology, Cell Biology and Genetics, Universidad de La Laguna, La Laguna, Santa Cruz de Tenerife, Spain.<br><br>CIBER de Enfermedades Respiratorias, Instituto de Salud Carlos III, Madrid, Spain.<br><br>Instituto de Tecnologías Biomédicas (ITB), Universidad de La Laguna, Santa Cruz de Tenerife, Spain.
Natalia Hernandez-Pacheco, MSc	Spain - Tenerife	Research Unit, Hospital Universitario N.S. de Candelaria, Universidad de La Laguna, Santa Cruz de Tenerife, Spain<br><br>Genomics and Health Group, Department of Biochemistry, Microbiology, Cell Biology and Genetics, Universidad de La Laguna, La Laguna, Santa Cruz de Tenerife, Spain.
Esther Herrera-Luis, MSc	Spain - Tenerife	Genomics and Health Group, Department of Biochemistry, Microbiology, Cell Biology and Genetics, Universidad de La Laguna, La Laguna, Santa Cruz de Tenerife, Spain.
Javier Perez-Garcia, BS	Spain - Tenerife	Genomics and Health Group, Department of Biochemistry, Microbiology, Cell Biology and Genetics, Universidad de La Laguna, La Laguna, Santa Cruz de Tenerife, Spain.
Fabian Lorenzo-Diaz, PhD	Spain - Tenerife	Genomics and Health Group, Department of Biochemistry, Microbiology, Cell Biology and Genetics, Universidad de La Laguna, La Laguna, Santa Cruz de Tenerife, Spain.
Uroš Potočnik, PhD	Slovenia	Center for Human Molecular Genetics and Pharmacogenomics, Faculty of Medicine, University of Maribor, Maribor, Slovenia.<br><br>Laboratory for Biochemistry, Molecular biology and Genomics, Faculty of Chemistry and Chemical Engineering, University of Maribor, Maribor, Slovenia.
Mario Gorenjak, MBI, PhD	Slovenia	Center for Human Molecular Genetics and Pharmacogenomics, Faculty of Medicine, University of Maribor, Maribor, Slovenia.

Katja Repnik, PhD	Slovenia	Center for Human Molecular Genetics and Pharmacogenomics, Faculty of Medicine, University of Maribor, Maribor, Slovenia.<br><br>Laboratory for Biochemistry, Molecular biology and Genomics, Faculty of Chemistry and Chemical Engineering, University of Maribor, Maribor, Slovenia
Vojko Berce,MD, PhD	Slovenia	Center for Human Molecular Genetics and Pharmacogenomics, Faculty of Medicine, University of Maribor, Maribor, Slovenia.<br><br>Clinic of Pediatrics, University Medical Centre Maribor, Maribor, Slovenia.
Michael Kabesch	Germany	Department of Pediatric Pneumology and Allergy, University Children's Hospital Regensburg (KUNO) at the Hospital St. Hedwig of the Order of St. John, University of Regensburg, Regensburg, Germany.<br>Science and Development Campus Regensburg (WECARE), University Children's Hospital Regensburg (KUNO) at the Hospital St. Hedwig of the Order of St. John, University of Regensburg, Regensburg, Germany.
Susanne Harner	Germany	Department of Pediatric Pneumology and Allergy, University Children's Hospital Regensburg (KUNO) at the Hospital St. Hedwig of the Order of St. John, University of Regensburg, Regensburg, Germany.
Christine Wolff	Germany	Science and Development Campus Regensburg (WECARE), University Children's Hospital Regensburg (KUNO) at the Hospital St. Hedwig of the Order of St. John, University of Regensburg, Regensburg, Germany
Antoaneta Toncheva	Germany	Department of Pediatric Pneumology and Allergy, University Children's Hospital Regensburg (KUNO) at the Hospital St. Hedwig of the Order of St. John, University of Regensburg, Regensburg, Germany.
Susanne Brandstetter	Germany	Science and Development Campus Regensburg (WECARE), University Children's Hospital Regensburg (KUNO) at the Hospital St. Hedwig of the Order of St. John, University of Regensburg, Regensburg, Germany.
Elisa Valletta	Germany	Science and Development Campus Regensburg (WECARE), University Children's Hospital Regensburg (KUNO) at the Hospital St. Hedwig of the Order of St. John, University of Regensburg, Regensburg, Germany.
Heike Buntrock-Döpke	Germany	Science and Development Campus Regensburg (WECARE), University Children's Hospital Regensburg (KUNO) at the Hospital St. Hedwig of the Order of St. John, University of Regensburg, Regensburg, Germany.

Joris C. Verster PhD	The Netherlands	Division of Pharmacology, Utrecht Institute for Pharmaceutical Sciences, Faculty of Science, Utrecht University, Utrecht, the Netherlands.<br><br>Institute for Risk Assessment Sciences, Faculty of Veterinary Medicine, Utrecht University, Utrecht, the Netherlands.
Nikki Kerssemakers	The Netherlands	Division of Pharmacology, Utrecht Institute for Pharmaceutical Sciences, Faculty of Science, Utrecht University, Utrecht, the Netherlands.
Catarina Almqvist MD, PhD	Sweden	Dept of Medical Epidemiology and Biostatistics, Karolinska Institutet, Stockholm, Sweden.<br><br>Pediatric Allergy and Pulmonology Unit at Astrid Lindgren Children's Hospital, Karolinska University Hospital.
Anna Hedman PhD	Sweden	Dept of Medical Epidemiology and Biostatistics, Karolinska Institutet, Stockholm, Sweden.
Mwenya Mubanga PhD	Sweden	Dept of Medical Epidemiology and Biostatistics, Karolinska Institutet, Stockholm, Sweden.
Tong Gong PhD	Sweden	Dept of Medical Epidemiology and Biostatistics, Karolinska Institutet, Stockholm, Sweden.
Anne Örtqvist	Sweden	Dept of Medical Epidemiology and Biostatistics, Karolinska Institutet, Stockholm, Sweden.

Others that contributed to SysPharmPediA:

Name:	Country:	Affiliation:
Amaia Lorea Alvarez RN	Spain	Division of Pediatric Respiratory Medicine, Hospital Universitario Donostia, San Sebastián, Spain.
Arantzazu Zugasti Pérez RN	Spain	Division of Pediatric Respiratory Medicine, Hospital Universitario Donostia, San Sebastián, Spain.
Miren Lorea Otero Aramburu, NA	Spain	Division of Pediatric Respiratory Medicine, Hospital Universitario Donostia, San Sebastián, Spain.
María Isabel Gomez Osua RN[3]	Spain	Basque Biobank (Basque Biobank), Biodonostia Health Research Institute Association Node www.biobancovasco.org
María Isabel Gomez Osua RN[3]	Spain	Basque Biobank (Basque Biobank), Biodonostia Health Research Institute Association Node www.biobancovasco.org
Yennece W.F. Dagelet	The Netherlands	Department of Respiratory Medicine, Amsterdam UMC, University of Amsterdam, Amsterdam, The Netherlands.
Suzanne Terheggen-Lagro MD, PhD	The Netherlands	Department of Pediatric Respiratory Medicine, Emma Children's Hospital, Amsterdam UMC, Amsterdam, The Netherlands.
Niels W.P. Rutjes MD	The Netherlands	Department of Pediatric Respiratory Medicine, Emma Children's Hospital, Amsterdam UMC, Amsterdam, The Netherlands.
E. G. Haarman	The Netherlands	Department of Pediatric Respiratory Medicine, Emma Children's Hospital, Amsterdam UMC, Amsterdam, The Netherlands.
T. Dekker	The Netherlands	Department of Experimental Immunology, Academic Medical Center (AMC), University of Amsterdam, Amsterdam, The Netherlands.
B.S Dierdorp	The Netherlands	Department of Experimental Immunology, Academic Medical Center (AMC), University of Amsterdam, Amsterdam, The Netherlands.
R. Khurshid	The Netherlands	Department of Experimental Immunology, Academic Medical Center (AMC), University of Amsterdam, Amsterdam, The Netherlands.
B. Kapitein, MD, PhD	The Netherlands	Paediatric Intensive Care, Emma Children's Hospital, Amsterdam UMC, Amsterdam, The Netherlands.
G. Biesbroek	The Netherlands	Department of Pediatric Respiratory Medicine, Emma Children's Hospital, Amsterdam UMC, Amsterdam, The Netherlands.
J. Israels	The Netherlands	Department of Pediatric Respiratory Medicine, Emma Children's Hospital, Amsterdam UMC, Amsterdam, The Netherlands.

C. Brackel	The Netherlands	Department of Pediatric Respiratory Medicine, Emma Children's Hospital, Amsterdam UMC, Amsterdam, The Netherlands.
M. Verwaal – Maasstad	The Netherlands	Department of Pediatrics, Maasstad Hospital, Rotterdam, The Netherlands.
D. Seljogi	The Netherlands	Department of Pediatric Respiratory Medicine, Emma Children's Hospital, Amsterdam UMC, Amsterdam, The Netherlands.
P.F.M. Mau Asam	The Netherlands	Department of Respiratory Medicine, Amsterdam UMC, University of Amsterdam, Amsterdam, The Netherlands.
S. J.A. Lone-Latif	The Netherlands	Department of Respiratory Medicine, Amsterdam UMC, University of Amsterdam, Amsterdam, The Netherlands.
Davey Stigters	The Netherlands	Utrecht University, Master Pharmacy, Utrecht, The Netherlands.
Emine Sariguney	The Netherlands	Department of Respiratory Medicine, Amsterdam UMC, University of Amsterdam, Amsterdam, The Netherlands.
André Franke	Germany	Kiel
Juha Kere	Sweden/Finland	Stockholm/Helsinki
Anne Örtqvist	Sweden	Dept of Medical Epidemiology and Biostatistics, Karolinska Institutet, Stockholm, Sweden.
Vilhelmina Ullemar	Sweden	Dept of Medical Epidemiology and Biostatistics, Karolinska Institutet, Stockholm, Sweden.
Karin Dellenvall	Sweden	Dept of Medical Epidemiology and Biostatistics, Karolinska Institutet, Stockholm, Sweden.

Funding:

The SysPharmPediA consortium is supported by ZonMW [project number: 9003035001], the Ministry of Education, Science, and Sport of the Republic of Slovenia [contract number C330-16-500106]; the German Ministry of Education and Research (BMBF) [project number FKZ 031L0088]; Instituto de Salud Carlos III (ISCIII) through Strategic Action for Health Research (AES) and European Community (EC) within the Active and Assisted Living (AAL) Program framework [award numbers AC15/00015 and AC15/00058] under the frame of the ERACoSysMed JTC-1 Call. M.P.-Y. was funded by the Ramón y Cajal Program (RYC-2015-17205) by the Spanish Ministry of Science and Innovation (MICINN), the State Research Agency, and the European Re-gional Development Fund from the European Union (MICINN/AEI/FEDER, UE, grant SAF2017-83417R). J.P.-G. was supported by a Ph.D. fellowship (FPU19/02175) granted by MICINN. U.P. and M.G. were funded by Slovenian Research Agency (research core funding No. P3-0067). M.I.A.-A. was funded by the Egyptian Government Ph.D. Scholarships. The STOPPA study was funded by the Swedish Research Council project grant 2018-02640 and the Swedish Asthma and Allergy Research Foundation.

Input Spain:

Human samples and related data used in this study were provided by the Basque Biobank/Biodonostia Node www.biobancovasco.org and were processed following standard operating procedures with appropriate approval of the relevant Ethics Committee and Scientific Advisory Board.
