# Supplementary figures and images for: A System Pharmacology Multi-Omics Approach toward Uncontrolled Pediatric Asthma
Source: J Pers Med. 2021 May 28;11(6):484. doi: 10.3390/jpm11060484 (PMC8227234; doi:10.3390/jpm11060484)

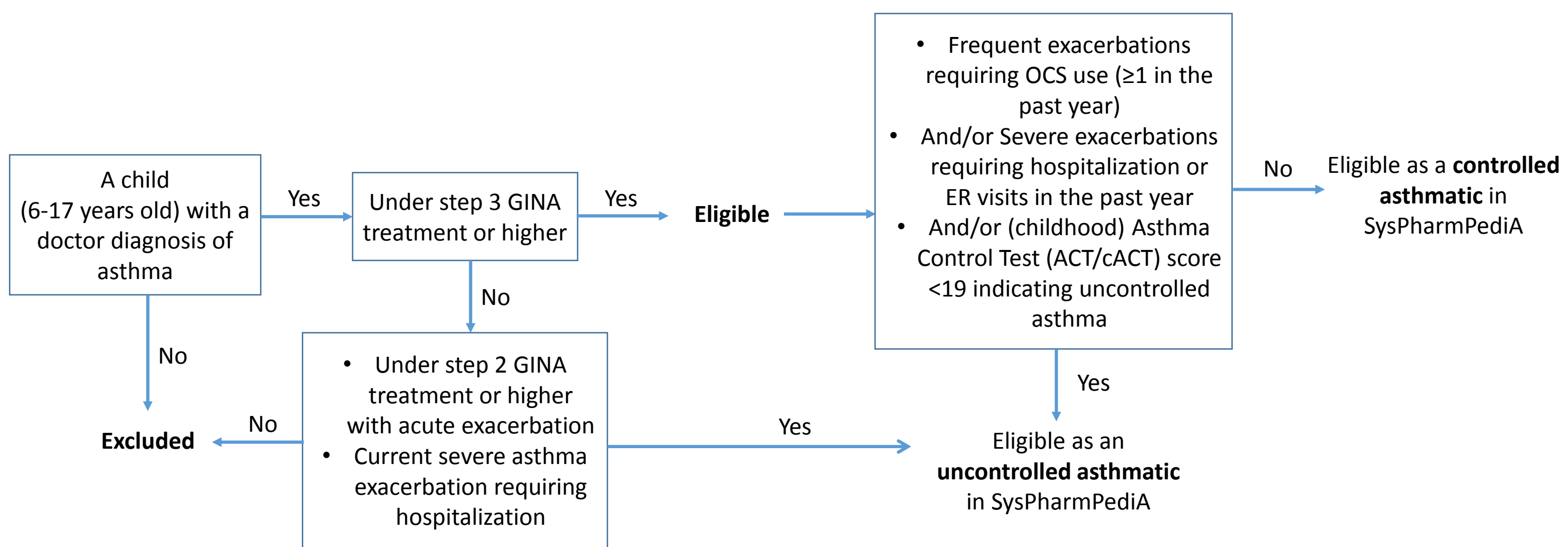

Supplement: Supplementary file 1 [file jpm-11-00484-s001.zip › Figure S1.pdf]
